# Fate of amnion-derived stem cells transplanted to the fetal rat brain: migration, survival and differentiation

**DOI:** 10.1111/j.1582-4934.2008.00180.x

**Published:** 2008-08-11

**Authors:** A J Marcus, T M Coyne, I B Black, D Woodbury

**Affiliations:** a The Ira B. Black Center for Stem Cell Research and the Department of Neuroscience and Cell Biology, University of Medicine and Dentistry of New Jersey-Robert Wood Johnson Medical SchoolPiscataway NJ, USA; b M.D./Ph.D Program, Graduate School of Biomedical Sciences, University of Medicine and Dentistry of New Jersey-Robert Wood Johnson Medical SchoolPiscataway, NJ, USA; c The Joint Graduate Program in Toxicology, Rutgers UniversityPiscataway, NJ, USA

**Keywords:** in utero transplantation, amniotic membrane, stem cells, differentiation, plasticity

## Abstract

We have recently characterized a stem cell population isolated from the rodent amniotic membrane termed amnion-derived stem cells (ADSCs). *In vitro* ADSCs differentiate into cell types representing all three embryonic layers, including neural cells. In this study we evaluated the neuroectodermal potential of ADSCs *in vivo* after *in utero* transplantation into the developing rat brain. A clonal line of green fluorescent protein-expressing ADSCs were infused into the telencephalic ventricles of the developing embryonic day 15.5 rat brain. At E17.5 donor cells existed primarily as spheres in the ventricles with subsets fused to the ventricular walls, suggesting a mode of entry into the brain parenchyma. By E21.5 green fluorescent protein (GFP) ADSCs migrated to a number of brain regions. Examination at postnatal time points revealed that donor ADSCs expressed vimentin and nestin. Subsets of transplanted ADSCs attained neuronal morphologies, although there was no immunohistochemical evidence of neural or glial differentiation. Some donor cells migrated around blood vessels and differentiated into putative endothelial cells. Donor ADSCs transplanted *in utero* were present in recipients into adulthood with no evidence of immunological rejection or tumour formation. Long-term survival may suggest utility in the treatment of disorders where differentiation to a neural cell type is not required for clinical benefit.

## Introduction

Stem cells have been touted as potential therapeutic agents for a wide spectrum of degenerative diseases from Parkinson's disease to lysosomal storage diseases. In recent years, extra-embryonic tissue, such as placenta [1, 2] and umbilical cord [3–6], have received a great deal of attention as alternative sources of multipotent stem cells for therapeutic use.

Extra-embryonic stem cells are particularly attractive for neural transplant strategies. In contrast to other adult tissues, the central nervous system and its resident neural stem cells (NSCs) demonstrate a limited capacity to regenerate diseased or injured tissue [7]. Moreover, the inaccessibility of NSC sources deep within the brain complicates their clinical exploitation. Therefore, transplantation of more easily accessible stem cell populations represents an alternative strategy to replace lost or injured neural elements. To address this need, we have initiated studies to investigate the neuroectodermal potential of stem cells derived from extra-embryonic tissue, specifically the amniotic membrane. The amniotic membrane and cells derived thereof are particularly appealing for allogeneic transplantation due to their immunoprivileged character [8, 9].

Recently, we have used a tissue explant method to isolate a stem cell population from the rat amniotic membrane, termed ADSCs. ADSCs are capable of extensive self-renewal and exist in culture in a multi-differentiated state, expressing neuroectodermal (nestin), meso-dermal (vimentin and fibronectin) and endodermal (α-1-antitrypsin) genes. *In vitro*, ADSCs differentiate into presumptive fat, bone and liver cells. In addition, ADSCs differentiate into putative neural cells *in vitro* after culture in a defined neural induction media [10]. Differentiated cells assumed neuronal morphologies and up-regulate neuron-specific genes, including tau and GAP43. To determine whether the neuroectodermal potential observed in culture correlates with their ability to differentiate into neural cell types in vivo, we have now transplanted green fluorescent protein (GFP)-expressing ADSCs into the rodent embryonic brain.

The developing brain is a fertile environment that contains a plethora of morphogenic cues that direct the temporal and regional development of endogenous cells, and may foster similar ontogenetic behaviours in donor cells. In fact, neural and even select non-neural stem cell populations are capable of responding to this complex environment, differentiating into neuroectodermal cell-types [11–15]. These studies suggest that the developing rodent brain is an ideal environment in which to assess the plasticity of adult and foetal stem cells. Using this model system, we now explore the plasticity of ADSCs *in vivo*, examining their potential to migrate, engraft, survive long-term, and differentiate into neuroectodermal cell types after *in utero* transplantation.

## Material and methods

### Animal welfare

Animal studies were performed in accordance with guidelines established by the Institutional Animal Care and Use Committee at UMDNJ-RWJMS.

### Reagents

All products were obtained from Sigma-Aldrich unless otherwise stated.

### ADSC isolation and culture

ADSCs were isolated and cultured as previously described [10]. Briefly, fragments of rat amniotic membrane, from embryonic day 18.5 (E18.5) Sprague-Dawley rat embryos were placed in 6-well plastic tissue culture dishes in a minimal volume (0.5 ml) of Dulbecco's modified Eagle's medium [DMEM, Invitrogen] supplemented with 20% Foetal Bovine Serum [FBS, Atlanta Biologicals]. Emergent cells were cultured as described.

### Generation of clonal lines

Clonal lines were generated from single cells as previously described [10, 16]. Briefly, ADSCs were plated at a density of 1 cell/cm^2^ in 150 mm plastic culture dishes and incubated for 2 hrs to allow cell attachment. Supernatant was removed and dishes were washed with phosphate buffered saline (PBS) to eliminate unattached cells. Dishes were examined microscopically and isolated single cells were identified and marked. Colonies arising from these single cells were then expanded into clonal lines.

### Neural Induction

Neuronal differentiation was performed as previously described [17] with modification. Briefly, GFP-expressing ADSCs cells were plated in 6-well plates at a density of 20,000 cells/well and treated 48 hrs after plating. Cells were rinsed three times with PBS, then transferred to neuronal induction media (NIM) containing 2 mM valproic acid, 15 mM betaine, 2.5 mM taurine, 175 μM butylated hydroxyanisole, 27 nM selenium, 20 nM progesterone, 10 μM forskolin, 10 nM K252a [Calbiochem], 5 units/ml heparin 5 μg/ml insulin, 1mM sodium pyruvate, 50 μM α thioglycerol, 20 nM bathocuproindisulfonic acid in a base of DMEM pH 7.0 (5.96 g/L HEPES, 2.5 g/L bicarbonate). The media was supplemented with 10 ng/ml basic fibroblast growth factor at 24 hrs after induction and every 48 hrs thereafter. Cells in neural induction media were maintained under a humidified atmosphere of 5% CO_2_ in air at 30°C for 7 days.

### Reporter vector construction and transfection into ADSCs

The pEF-1/eGFP vector used for transfection of ADSCs was generated from two commercially available vectors. pEf1/myc/his [Invitrogen] containing the elongation factor 1 promoter (EF1) upstream of a multiple cloning site (MCS) served as the vector backbone. The eGFP gene, isolated from pVivo2-GFP-LacZ [InvivoGen] was inserted into the MCS, allowing regulated expression by the EF1 promoter. The EF1-GFP chimeric vector was introduced into late passage (approximately passage 20) ADSCs using Lipofectamine [Invitrogen] as recommended by the manufacturer. Stable transfectants were selected for with G418. Clonal lines expressing high levels of GFP were identified *via* microscopic examination. For transplantation studies we utilized GFP-ADSC clone 2, which demonstrated the stem cells characteristics of self-renewal and multi-differentiation *in vitro*[10]. This clone uniformly expressed GFP and stable reporter gene expression was maintained at least 25 passages, the longest time examined.

### Transuterine intraventricular injection

Timed-pregnant Sprague Dawley rats, 15.5 days post-coitum (E15.5) served as hosts. Four animals were analysed per time point. Animals were sedated by an i.p. injection of ketamine (50 mg/kg) xylazine (2.6 mg/kg) acepromazine (0.65 mg/kg). A 3 cm ventral midline incision exposed the abdominal cavity, revealing the uterine horns and enclosed embryos. Guided by fibre optic transillumination, 2–3 μl of ADSCs cell suspension (100,000–150,000 cells in total) were pressure-injected into the lateral ventricles of foetal brains using a glass capillary pipette. Successful injections were evidenced by rapid diffusion of the fast green dye throughout the ventricular system. Embryo mortality as result of infusion was <5%. All surviving animals contained donor cells. Embryos that did not receive donor cells, or in which diffusion of dye throughout the ventricular system was not evident, were sacrificed in utero by intraventricular infusion of 9% saline.

### Tissue processing

For prenatal time points, dams were euthanized, embryos were retrieved and brains were microdissected. Brain tissue was immersion fixed in 4% paraformaldehyde (PFA) for 24 hrs at 4°C, rinsed 3× with PBS, then fixed for an additional 24 hrs in 30% sucrose/4% PFA. Tissue was then stored in 30% sucrose/PBS until processing. Postnatal animals were euthanized by an injection of pentobarbital (0.5 ml, 50 mg/ml), perfused with saline, followed by 4% PFA. Brains were removed and post-fixed in 4% PFA for 24 hrs, and subsequently stored in 30% sucrose/PBS until they were sectioned. All samples were sagitally, coronally or horizontally cryosectioned at 16 μm, and processed immunohistochemically.

### Immunohistochemistry and microscopic analysis

Slides containing 16 μm sections were rinsed extensively with PBS. Slides were placed in citric acid buffer solution (pH 6.0), microwaved until boiling and allowed to cool slowly to ambient temperature. The microwave step was repeated three times for optimal antigen retrieval. Tissues were blocked with 5% donor goat serum for 45 min. Sections were subsequently incubated for 24 hrs with primary antibodies: GFP ([mouse, rabbit], 1:250–1:500, Chemicon), vimentin ([mouse, rabbit], 1:250–1:500, Santa Cruz Biotechnologies), Nestin ([mouse], 1:250, DSHB), ß-III-tubulin ([mouse], 1:500, Chemicon), glial fibrillary acidic protein (GFAP) ([rabbit], 1:1000, Sigma), NeuN ([mouse], 1:250, Chemicon), MAP2 ([rabbit], 1:250, Chemicon), adenomatous polyposis coli (APC) ([mouse], 1:200, Calbiochem), von Willebrand factor (vWF) ([rabbit], 1:500, Chemicon) and ED1 ([ms], 1:100; Serotec Ltd.). After numerous washes, the sections were incubated with secondary antibodies: species-specific Alexa Fluor 594 or 488 was used (1:500, Molecular Probes). 4,6-Diamidino-2-phenyindole (DAPI) [1 μg/ml] or propidium iodide (PI) [20 ug/ml dH20] were used as nuclear counterstains. Negative controls were performed by omitting the primary antibody. All samples were cover slipped with Fluoromount G [Electron Microscopy Systems] and visualized with an inverted fluorescent microscope [Zeiss Axiovert]. Z-sectioning was utilized to co-localize all cellular markers.

## Results

### *In vitro* characterization of ADSCs

**Fig. 1:**
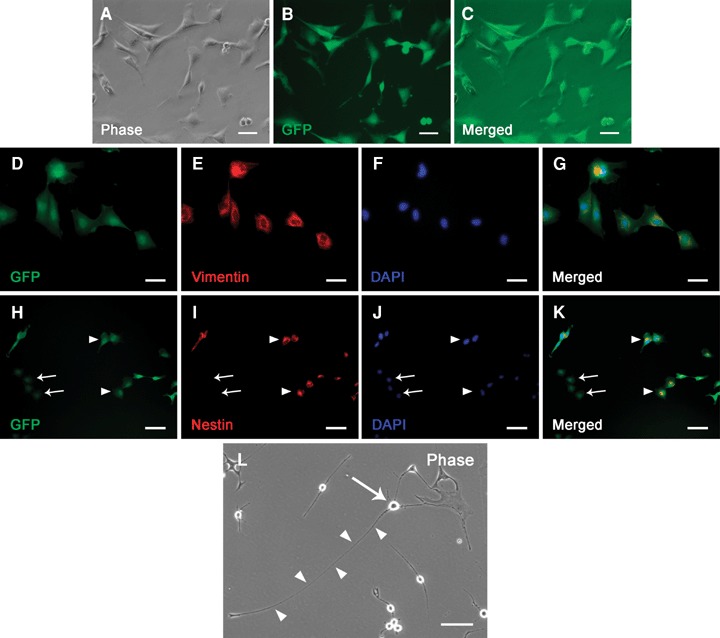
Characterization of green fluorescent protein (GFP)-expressing amnion-derived stem cells (ADSCs) prior to transplantation. (**A**-**C**) Examination of the same field with phase contrast and fluorescent microscopy reveals uniform expression of GFP in ADSCs. Regardless of the passage number close to 100% of the cells expressed GFP. (**D**-**G**) GFP-expressing ADSCs expressed the mesodermal marker vimentin, while a subset of cells (**H**-**K**, arrowheads) expressed the neural progenitor marker nestin. (**H**-**K**) The majority of ADSCs (arrows) were negative for nestin. (**L**) After culture in a defined neural induction media GFP^+^ ADSCs attained a neural morphology, exhibiting round, compact, refractile cell bodies (arrow), while elaborating long processes extending more than 600 μm in length (>'s). Scale Bars: A-C = 50 μm; D-G = 100 μm; H-L = 100 μm

For transplantation studies we utilized a clonal population of ADSCs which uniformly expressed GFP (Fig. 1A–C). This clonal population demonstrated the stem cell characteristics of self-renewal and multi-potency, including the ability to differentiate into neuron-like cells *in vitro*[10]. Close to 100% of the cells expressed vimentin (Fig. 1D–G), while a subset expressed the neural progenitor marker nestin. (Fig. 1H–K, arrowheads). Overexpression of the GFP gene did not affect the cells ability to respond morphologically to the *in vitro* neural induction protocol (Fig. 1L).

### Transplantation of GFP-expressing ADSCs into the embryonic brain

To determine whether ADSCs assume neuronal functions *in vivo* and assess potential use for cellular therapy, we characterized donor cells after transplantation into the embryonic day 15.5 (E15.5) rat brain. 1–1.5 × 10^5^ donor GFP-expressing cells suspended in 2–3 μl of DMEM were injected into the telencephalic ventricles of each recipient embryo. Invasion, migration, localization, phenotypic expression and long-term survival were assessed at various times after transplantation.

**Fig. 2:**
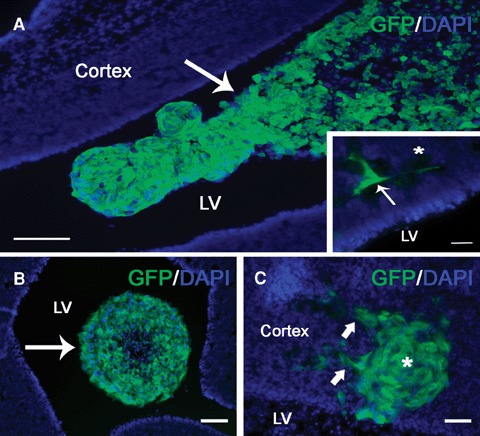
Infused ADSCs dispersed throughout the embryonic ventricular system and formed discrete spheres. (**A**) Sagittal section revealed extensive diffusion of donor ADSCs (arrow) throughout the ventricular system at E16.5 (24 hrs post-transplantation). (**A**, inset, arrow) Some donor cells were observed within the parenchyma of the brain even at this early time point. (**B**) By E17.5 donor cells formed discrete GFP^+^ spheres or clusters (arrow) within the lateral ventricles. (**C**) Some spheres appeared to have fused with the walls of the ventricular cavity. Individual cells (arrows) could be observed migrating into the parenchyma. LV = lateral ventricle, *= cortex. Scale bars: A, B = 100 μm; A- inset = 20 μm; C = 50 μm

We initially characterized short-term survival and engraftment 24 hr after transplantation. At E16.5 donor cells were diffused throughout the ventricular system (Fig. 2A). Some cells could also be identified within the parenchymal of the brain at this early time point (Fig. 2A, inset). By E17.5, discrete spherical clusters consisting of GFP+ cells were observed within the ventricles (Fig. 2B, arrow). A few clusters appeared to have fused with the walls of the ventricles (Fig. 2C) and individual donor cells could be seen in the brain parenchyma (Fig. 2C, arrows). Widespread distribution of transplanted ADSCs was detected at later time points.

**Fig. 3:**
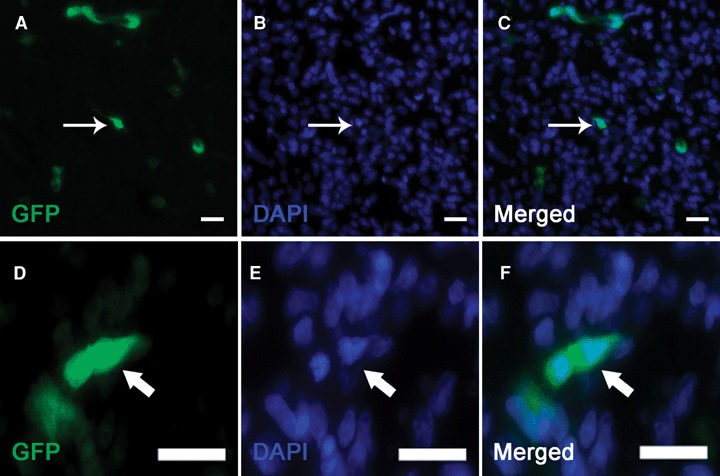
Distribution of ADSCs at E20.5. (**A**-**C**) At E20.5 GFP^+^ ADSCs (arrow) were observed in a number of brain areas, including the cortex. (**D**-**F**) Donor ADSCs (arrow) were also evident in the midbrain. Scale bars: **A**-**F**= 20 μm.

At E20.5, donor cells were consistently observed in multiple brain areas, including the cortex (Fig. 3A–C, arrow) and midbrain (Fig. 3D–F, arrow). Distribution of GFP^+^ cells at later postnatal time points seemed to be random- varying from animal to animal. In some animals cells could be localized to the hippocampus and striatum while in others they were observed in the thalamus (data not shown).

### Phenotypic characterization of transplanted ADSCs

**Fig. 4:**
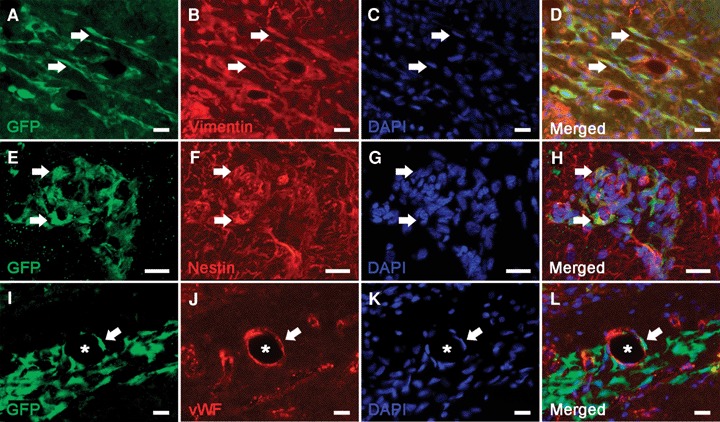
Morphology and Phenotypic characterization of transplanted ADSCs at 1 week postnatally. (**A**-**D**) The majority of transplanted GFP^+^ ADSCs (arrows) within the cortex attained elongated morphologies and expressed vimentin. (**E**-**H**) Some donor cells (arrows) were nestin positive and assumed ameboid morphologies. (**I**−**J**) A sub-population of donor cells (arrow) around blood vessels attained crescent morphologies and expressed von Willebrand factor (vWF). *= blood vessel. Scale bar: A-L = 20 μm.

The majority of donor cells that migrated into the parenchyma by P7, or 1 week postnatally, did not integrate within the normal cytoarchitecture of the brain. In most cases, the ADSCs assumed either elongated (Fig. 4A, arrows) or ameboid (Fig. 4E, arrows) morphologies. Mirroring the expression pattern in culture, the majority of ADSC expressed vimentin (Fig. 4A–D, arrows), while a subset also expressed Nestin (Fig. 4E–H, arrows). Interestingly, a sub-population of cells that migrated around blood vessels attained crescent morphologies and expressed vWF (Fig. 4I–L, arrow), a blood glycoprotein involved in coagulation. These morphologic and phenotypic observations are consistent with an endothelial cell differentiation of donor ADSCs. None of the examined cells showed evidence of multiple nuclei, suggesting that fusion with endogenous cells did not contribute to donor cell survival or differentiation.

**Fig. 5:**
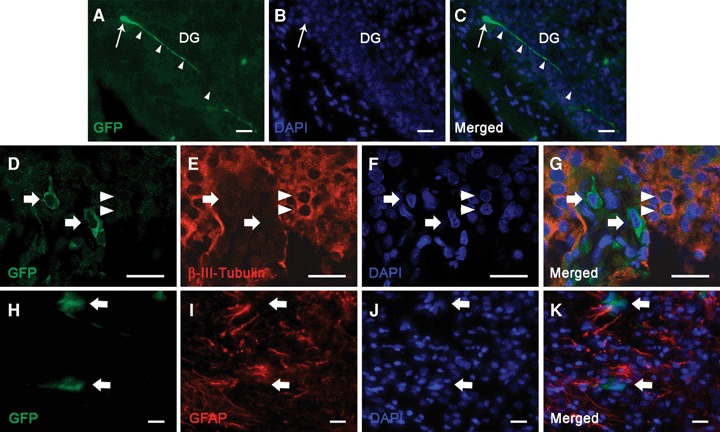
A sub-population of transplanted ADSCs assumed neuronal morphologies. (**A**−**C**) At P7, a subset of GFP^+^ cells in the hippocampal formation assumed typical neural morphologies, small cell bodies (arrow) and the elaboration of long processes (arrowheads). (**D**−**G**) Donor cells (arrows) within the cortex did not express the neuronal protein, β-III-tubulin, in contrast to neighbouring endogenous cells (arrowheads). (**H**−**K**) GFP^+^ ADSCs did not express the astrocyte structural protein GFAP DG = Dentate Gyrus. Scale bar: **A**-**K**= 20 μm.

Some ADSCs that engrafted in the brain did attain neuronal morphologies- small cell body with long process extensions (Fig 5A–C, arrow). Despite these morphological changes, no donor cells were found to up-regulate any neural or glial markers, including b-III-Tubulin (Fig. 5D–G, arrows) and GFAP (Fig. 5H–K, arrows). Transplanted ADSCs were also negative for the mature neural markers MAP2, NeuN and the oligodendrocyte marker APC (data not shown).

### Long-term survival of donor cells

**Fig. 6:**
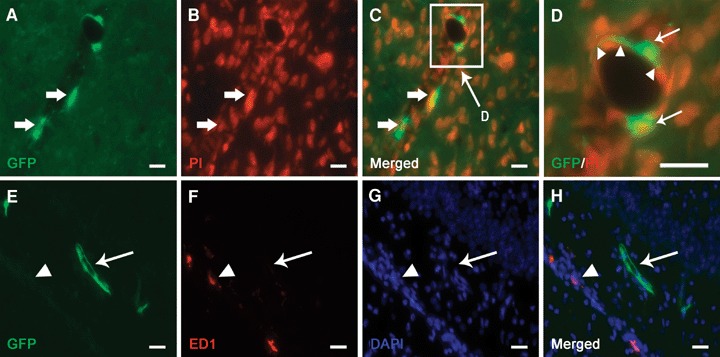
Long-term survival of transplanted ADSCs in the neocortex. (**A**−**C**) In utero transplanted ADSCs (arrows) survive 2.5 months in the neocor-tex of recipient animals. (**D**) Higher magnification of the box in (**C**) reveals that a subset of donor cells (arrows) in the adult brain associated with vascular structures. (**E**−**H**) GFP^+^ ADSCs in the P7 rat hippocampus did not initiate a host immunological response as indicated by the absence of ED1 ^+^ reactive microglia within the proximity of donor cells (arrow). ED1^+^ (**E**−**H**, arrowhead) are present, but not closely associated with donor GFP ADSCs. PI = Propidium Iodide. Scale bar: A-H = 20 μm.

Even in the absence of neuronal differentiation, donor ADSCs were able to survive in cortical (Fig. 6A–C, arrows) and vascular regions (Fig. 6D, arrow) up to two and half months postnatally, the longest time examined. No evidence of a host inflammatory response or immunological rejection was observed, at early (P7) or later postnatal time points, as indicated by the absence of ED1^+^ reactive microglia cells (Fig. 6E–H).

## Discussion

### Overview

We have previously described the characterization of a stem cell population isolated from the rat amniotic membrane [10]. These ADSCs are able to undergo extensive self-renewal and differentiate *in vitro* into cells representing all germinal layers, including the neuroectoderm. In this report we characterize these cells after transplantation into the embryonic brain at a time when active neurogenesis is occurring.

### Donor ADSCs in the embryonic brain: sphere formation, invasion and migration

Numerous studies have demonstrated the feasibility of using the developing brain as a tool for assessing stem cells plasticity in vivo [11–15, 18–20]. We have exploited this model system, transplanting GFP-expressing ADSCs into the telencephalic ventricles of the developing E15.5 rat brain. We observed that donor ADSCs form spherical clusters within the ventricles within 48 hrs of transplantation. These cellular spheres then fused with the periventricular areas of the brain parenchyma and individual cells began to migrate to various regions of the brain. This method of entry into the brain may be unique to stem cell populations. Brustle et al. demonstrated that NSCs cluster together and form discrete spheres prior to invading the brain parenchyma [11]. In another study Munoz-Elias *et al*. showed that adult bone marrow stromal cells transplanted into the developing rat brain form intraventricular spheres prior to fusing with the walls of the ventricles [13]. Sphere formation may facilitate access to the brain parenchyma. Recent experiments in our lab demonstrate that GFP-expressing fibroblasts transplanted into the embryonic brain displayed no cluster formation and were not observed to migrate into the brain (data not shown). However, Coenen *et al*. reported that human umbilical cord blood-derived adherent progenitors transplanted into the developing rat brain form spheres, but remain largely confined to the ventricles. Clusters of donor cells survived for up to 4 weeks after transplantation within the ventricles but did not migrate or engraft within the brain parenchyma, suggesting that sphere formation alone is not sufficient to allow engraftment. The authors indicated that this study may have been complicated by the transplantation of human cells into the rodent brain. Perhaps sites of engraftment exploited by donor rodent ADSCs in the current study were not recognized by human umbilical cord blood-derived adherent progenitors.

### ADSC differentiation and long-term survival

Within the brain donor ADSCs do not appear to respond to local neurogenic cues. Surviving ADSCs did obtain neural morphologies in some instances, but did not appear to integrate within the normal cytoarchitecture of the brain. Expression of neural markers by donor cells was not detected, even at advanced postnatal times. This is in contrast to the fate of other stem cell populations examined using this model system, including adult bone marrow stromal cells [13] and human umbilical cord blood cells [20], both of which differentiate into neuroectodermal phenotypes subsequent to transplantation into the developing neurogenic rat brain. We hypothesize that *in vitro* pre-differentiation of donor ADSCs might be required to facilitate neural differentiation in vivo.

This study demonstrates that ADSCs are able to migrate into the brain and engraft in a number of brain regions. Donor ADSCs survived at least two and a half months postnatally, with no evidence of immunological rejection. Tolerance of allogeneic donor cells may be due to their inherent immunoprivileged state, or the immunological permissiveness of the developing brain. While the lack of neural differentiation was disappointing, this does not necessarily preclude the utility of ADSCs for the treatment of neurological disorders. Recent evidence suggests that overt differentiation of transplanted stem cells to a neural cell-type is not a prerequisite for therapeutic utility. Undifferentiated stem cells may be therapeutic through an alternative ‘by-stander’ pathway, perhaps involving the secretion of neurotrophins, chemokines and cytokines, immunomodulation or stimulation of host repair mechanism [21–23]. This effect does not seem to be limited to the brain; functional improvement in the absence of differentiation has been reported in animal models of diabetes, lung and cardiovascular disease [23–26].

The long-term expression of transfected GFP in donor cells suggests that genetically modified ADSCs may be useful for delivering therapeutic molecules to treat disease. In fact, stem cells have been used in animal models to treat a wide range of disorders, from lysosomal storage diseases to neural tube defects [27, 28]. A recent clinical trial by Le Blanc *et al.* has suggested that *in utero* therapy with foetal mesenchymal stem cells could be used to treat osteogenesis imperfecta, a disease once considered therapeutically unapproachable [29]. In light of these advances, *in utero* transplantation of ADSCs into the developing brain might lead to the development of numerous treatment paradigms for congenital neurological disorders.

Although the majority of transplanted cells remained undifferentiated, some donor ADSCs did respond to local vascular niches and differentiated into presumptive endothelial cells. Prior to transplantation ADSCs express both mesodermal and ectodermal genes. The results from our current study suggest that these cells are mesodermal-like at their core and are therefore able to respond appropriately to mesodermal signals, in this case those associated with blood vessels. If pushed toward other lineages *in vitro*, prior to transplantation, ADSCs might be able to respond to *in vivo* ectodermal and endodermal cues as well. *In vivo* endothe-lial differentiation of ADSCs may indicate their suitability for the treatment of adult onset diseases in which the growth of new blood vessels is desired, including cerebrovascular and cardiovascular disease.

## Conclusions

In summary, this study demonstrates that ADSCs transplanted *in utero* survive into early adulthood, with no evidence of rejection or tumour formation. Differentiation to neural lineages was not observed, but donor cells appeared competent for endothelial differentiation. Importantly, ADSCs demonstrate long-term survival and maintain expression of a transgene in the absence of ongoing selection. ADSCs may therefore be useful vectors for the delivery of therapeutic proteins to the central nervous system where neural differentiation is not a prerequisite. As an example, ADSCs may be of clinical benefit in the treatment of lysosomal storage diseases, a contention we are currently investigating.
